# The Role of Emotion Regulation in Trauma Processing During Eye Movement Desensitization and Reprocessing (EMDR) Therapy

**DOI:** 10.3390/healthcare14183107

**Published:** 2026-09-20

**Authors:** Francisco J. Gómez-Salas, Alejandra Ramallo-Machín, Francisco Burgos-Julián, M. A. Santed-Germán, Ana Isabel Gonzalez-Vazquez

**Affiliations:** 1 Facultad de Psicología y Logopedia, Universidad de Málaga, Andalucía Tech, Campus de Teatinos s/n, 29071 Málaga, Spain; 2 Facultad de Psicología y Ciencias de la Salud, Universidad a Distancia de Madrid (UDIMA), 28400 Madrid, Spain; 3 Facultad de Ciencias, Universidade da Coruña (UDC), Campus da Zapateira, 15071 A Coruña, Spain; alejandra.ramallo.machin@udc.es; 4 Facultad de Psicología, Universidad Nacional de Educación a Distancia (UNED), 28040 Madrid, Spain; msanted@psi.uned.es; 5 Departamento de Psiquiatría, Complexo Hospitalario Universitario de A. Coruña, Xubias de Arriba 1, 15006 A Coruña, Spain; anabel.gonzalez.vazquez@gmail.com

**Keywords:** Eye Movement Desensitization and Reprocessing, emotional regulation, psychological trauma, rumination, cognitive, psychotherapeutic processes

## Abstract

**Background/Objectives**: Emotion regulation may be relevant to trauma processing during Eye Movement Desensitization and Reprocessing (EMDR) therapy, but associations between specific regulatory processes and distinct processing patterns remain unclear. This study examined these associations and cross-sectional indirect associations with symptom severity. **Methods**: This multicenter cross-sectional study included 116 patients receiving EMDR therapy between 2023 and 2025. Patients completed measures of emotion regulation, emotional processing, rumination, alexithymia, childhood trauma, and psychological symptom severity; therapists completed the Processing Difficulties Scale (PDS). Analyses included Pearson correlations with Benjamini–Hochberg false discovery rate correction, multivariable linear regression, and bootstrap cross-sectional indirect-effect analyses. **Results**: Overall emotion regulation difficulties were not significantly associated with any PDS dimension, whereas specific processes showed distinct bivariate associations. In multivariable linear regression models, lower emotional awareness was independently associated with greater Lack of Generalization and/or Absence of Changes (β = −0.215, *p* = 0.028), hypothetical thinking with greater Poor Emotional Processing (β = 0.235, *p* = 0.011), and more EMDR processing sessions with greater Loss of Dual Attention (β = 0.264, *p* = 0.005). Models explained 5.5–18.4% of the variance. Lower emotional awareness showed a significant cross-sectional indirect association with symptom severity through Lack of Generalization and/or Absence of Changes. **Conclusions**: Specific emotional-cognitive processes were differentially associated with trauma-processing patterns during EMDR. The cross-sectional design and modest explanatory capacity of the models preclude causal or temporal interpretations. Prospective studies are needed to assess the predictive and clinical utility of the PDS.

## 1. Introduction

Psychological trauma has increasingly been conceptualized as a transdiagnostic risk factor for a wide range of psychiatric disorders, including posttraumatic stress disorder (PTSD), dissociative disorders, personality disorders, mood disorders, and anxiety disorders [1,2,3,4]. Accumulating evidence suggests that traumatic experiences, particularly when occurring early in life and repeatedly over time, are associated with persistent alterations in emotion regulation, cognitive processing, stress-response systems, and interpersonal functioning, which may contribute to shared vulnerability across multivariable forms of psychopathology and a broad range of chronic medical conditions in adulthood [2,5,6,7,8].

Within this framework, emotion regulation has emerged as an important transdiagnostic process associated with both trauma exposure and psychopathology [2]. Emotion regulation refers to the capacity to monitor, evaluate, and modify emotional responses across physiological, experiential, cognitive, and behavioral domains [9], and difficulties in these processes have been consistently associated with greater psychological distress and functional impairment [10]. However, emotion regulation is not a unitary construct. Contemporary models increasingly emphasize the importance of distinguishing broad difficulties in emotion regulation from specific regulatory strategies and related emotional-cognitive processes, including emotional suppression, experiential avoidance, rumination, alexithymia, and deficits in emotional awareness and clarity [11,12,13,14,15,16,17,18]. These processes are conceptually distinct and may show differential associations with trauma-related symptoms and emotional processing. This perspective is consistent with process-based approaches to psychotherapy, which emphasize the identification of specific processes of change beyond broad diagnostic categories [14,19,20]. However, whether these specific regulatory and emotional-cognitive processes are differentially associated with patterns of trauma processing during EMDR therapy remains unclear.

Contemporary theoretical models emphasize emotional processing and the adaptive updating of emotional memories as processes relevant to therapeutic change [21,22,23,24,25,26]. Within this framework, emotional and cognitive processes have been proposed to shape how traumatic memories are accessed, experienced, and integrated during trauma-focused psychotherapy [21,23].

Eye Movement Desensitization and Reprocessing (EMDR) therapy has been extensively validated as an evidence-based treatment for PTSD [27,28,29]. According to the Adaptive Information Processing (AIP) model, EMDR therapy is proposed to facilitate the reprocessing of traumatic memories that are dysfunctionally stored and insufficiently integrated within adaptive memory networks, with such memories hypothesized to contribute to the persistence of trauma-related psychological symptoms [28].

EMDR therapy follows an eight-phase protocol that includes case conceptualization and treatment planning (Phase 1), preparation (Phase 2), and the assessment and reprocessing of traumatic memories (Phases 3–8) [29,30]. Within the AIP framework, adaptive processing involves the integration of emotional experiences, updating of maladaptive meanings, and incorporation of adaptive information into existing memory networks [28].

In clinical practice, considerable variability is observed in how patients process traumatic memories during EMDR memory reprocessing [29]. Some patients show fluent associative processing, emotional integration, and adaptive shifts in the meaning of traumatic experiences, whereas others exhibit emotional blocking, cognitive avoidance, perseverative associative processing, dissociative phenomena, or difficulties maintaining dual attention. Dual attention (the capacity to remain engaged with the traumatic memory while maintaining orientation to the present) is a characteristic feature of EMDR memory reprocessing [28,31,32].

In particular, rumination involves repetitive and perseverative thinking that may reduce cognitive flexibility and maintain attention on trauma-related distress [33,34]. Emotional suppression, in contrast, involves attempts to inhibit emotional expression or responding and has been associated with altered emotional processing and heightened physiological responses to stress [35,36]. Experiential avoidance refers more broadly to efforts to escape or reduce contact with distressing internal experiences, which may restrict engagement with emotionally salient material [15,37,38]. Alexithymia is characterized by difficulties identifying and describing emotional states and may therefore be relevant to their differentiation and elaboration [39,40,41,42].

In the treatment of complex trauma, the extent of preparation or stabilization needed before trauma-focused processing remains debated [43,44,45]. Although this debate has largely focused on treatment outcomes, examining how individual emotional and cognitive characteristics are associated with trauma processing during therapy may provide a complementary process-oriented perspective [23].

Recently, Ramallo-Machín et al. developed the Processing Difficulties Scale (PDS), a clinician-rated measure designed to operationalize differences in trauma processing during EMDR therapy [46]. The PDS comprises four dimensions reflecting different patterns of trauma processing: Good Processing, Lack of Generalization and/or Absence of Changes, Poor Emotional Processing, and Loss of Dual Attention. Initial validation supported its four-factor structure and showed good-to-excellent internal consistency across dimensions [46]; however, as a recently developed clinician-rated measure, further evidence regarding interrater reliability, predictive validity, and sensitivity to therapeutic change is needed.

Despite extensive research linking emotion regulation to symptom severity [47,48,49,50,51,52], comparatively little is known about its associations with specific patterns of trauma processing during EMDR. Characterizing these associations may help generate hypotheses about emotional and cognitive factors relevant to trauma processing and inform future research on individualized preparation and therapeutic support during EMDR.

Accordingly, the present study aimed to examine whether specific regulatory and related emotional-cognitive processes are differentially associated with patterns of trauma processing in a clinical sample of patients receiving EMDR therapy. Specifically, we investigated the associations between general difficulties in emotion regulation, specific regulatory and related emotional-cognitive processes (i.e., rumination, emotional suppression, avoidance, and alexithymia), and the four dimensions of trauma processing assessed with the Processing Difficulties Scale (PDS). We further explored cross-sectional indirect associations between these processes and overall symptom severity through the PDS dimensions, without assuming temporal or causal ordering.

## 2. Materials and Methods

### 2.1. Study Design

A multicenter, cross-sectional study was conducted to examine the associations between emotion regulation and related emotional-cognitive processes and patterns of trauma processing during Eye Movement Desensitization and Reprocessing (EMDR) therapy in a clinical sample of patients receiving EMDR treatment. Participants were recruited through participating therapists and clinical centers across 17 Spanish provinces, as well as in Argentina and Chile.

All participating therapists were EMDR Clinicians and treated patients using the standard EMDR therapy protocol. At the time of assessment, all patients were actively engaged in trauma memory reprocessing. The number of EMDR sessions recorded in the present study referred specifically to sessions in which trauma memory reprocessing was conducted, rather than to the total number of psychotherapy sessions received.

### 2.2. Participants

Following the application of the inclusion and exclusion criteria and completion of the questionnaires required for the study, 116 patients constituted the final analytical sample. All participants had provided written informed consent to participate in the study.

Psychiatric diagnoses were assessed using the Mini-International Neuropsychiatric Interview-Plus (MINI-Plus) [53]. The inclusion criteria were as follows: (1) meeting diagnostic criteria for at least one mental disorder according to the MINI-Plus; (2) being between 18 and 65 years of age; (3) having received EMDR therapy for at least three months; and (4) having the legal capacity to provide written informed consent to participate in the study.

The exclusion criteria were as follows: (1) the presence of a severe and unstable medical condition; (2) inability to understand and complete the study questionnaires; and (3) lack of adherence to psychotherapy.

### 2.3. Recruitment and Measures

A total of 228 participants were initially recruited between 2023 and 2025 through participating therapists and clinical centers located across 17 Spanish provinces, as well as in Argentina and Chile. Of these, 116 participants completed the questionnaires required for the present study and constituted the final analytical sample. Participants were treated by 13 therapists, with a median of 5 patients per therapist (range = 1–30).

The study was approved by the Research Ethics Committee of A Coruña-Ferrol (Galicia, Spain; approval code: 2017/425 EPTE). All participants provided written informed consent prior to enrollment in the study. The study was conducted in accordance with the ethical principles of the Declaration of Helsinki.

Participants completed a battery of standardized self-report questionnaires assessing general difficulties in emotion regulation, specific regulatory and related emotional-cognitive processes (i.e., rumination, emotional suppression, avoidance, and alexithymia), childhood trauma, and general psychological symptom severity. In addition, therapists completed the Processing Difficulties Scale (PDS) to assess patterns of trauma processing during EMDR therapy. The assessment instruments used in the present study are described below:

1. Processing Difficulties Scale (PDS). The Processing Difficulties Scale (PDS) is a clinician-rated instrument designed to assess dimensions of trauma processing during Phases 3–8 of the standard EMDR therapy protocol [46]. The instrument is completed retrospectively by the EMDR therapist responsible for the patient’s treatment, based on repeated clinical observations across multiple therapy sessions. It consists of 17 items rated on a five-point Likert scale (0 = Never to 4 = Always) and comprises four dimensions: Good Processing, Lack of Generalization and/or Absence of Changes, Poor Emotional Processing, and Loss of Dual Attention. Higher scores on each subscale indicate a greater presence of the corresponding processing pattern during trauma memory reprocessing. The original validation study supported a four-factor structure explaining approximately 55% of the total variance, with satisfactory model fit indices (RMSEA = 0.07, TLI = 0.91, CFI = 0.95) and good-to-excellent internal consistency across the four dimensions (Cronbach’s α = 0.82–0.92; McDonald’s ω = 0.83–0.94) [46]. In the present sample, internal consistency was good to excellent across the four PDS dimensions (F1: α = 0.94, ω = 0.94; F2: α = 0.88, ω = 0.89; F3: α = 0.81, ω = 0.82; F4: α = 0.85, ω = 0.86).
2. Difficulties in Emotion Regulation Scale (DERS). Overall difficulties in emotion regulation were assessed using the Difficulties in Emotion Regulation Scale (DERS) [12]. The validated Spanish version developed by Hervás and Jódar [54] was administered. This version comprises 28 items rated on a five-point Likert scale ranging from 1 (Almost Never) to 5 (Almost Always) and assesses five dimensions of emotion regulation difficulties: Emotional Dyscontrol, Interference in Daily Functioning, Emotional Inattention, Emotional Confusion, and Emotional Rejection. Higher scores indicate greater difficulties in emotion regulation. The Spanish version has demonstrated excellent internal consistency for the total score (Cronbach’s α = 0.93) [54]. Both the total score and the five subscale scores were included in the statistical analyses.
3. Emotional Processing Scale (EPS-25). Emotional processing was assessed using the Emotional Processing Scale (EPS-25) [55]. The scale comprises 25 items rated on a 10-point Likert scale ranging from 0 (Completely Disagree) to 9 (Completely Agree) and assesses five dimensions of emotional processing: Suppression, Signs of Unprocessed Emotion, Unregulated Emotion, Avoidance, and Impoverished Emotional Experience. Higher scores indicate greater difficulties in emotional processing. The EPS-25 has demonstrated high internal consistency for the total score (Cronbach’s α = 0.94) [55]. The five EPS-25 subscale scores were included in the statistical analyses.
4. Toronto Alexithymia Scale (TAS-20). Alexithymia was assessed using the Toronto Alexithymia Scale (TAS-20) [56], employing the Spanish adaptation developed by Martínez-Sánchez [57]. The TAS-20 is a 20-item self-report questionnaire rated on a five-point Likert scale ranging from 1 (Strongly Disagree) to 5 (Strongly Agree). It assesses three dimensions of alexithymia: Difficulty Identifying Feelings, Difficulty Describing Feelings, and Externally Oriented Thinking. Higher total scores indicate higher levels of alexithymia. The Spanish version has demonstrated satisfactory internal consistency (Cronbach’s α = 0.73) and good test–retest reliability [57]. The three TAS-20 subscale scores were included in the statistical analyses.
5. Ruminative Thought Style Questionnaire (RTSQ). Rumination was assessed using the Ruminative Thought Style Questionnaire (RTSQ) [58], employing the validated Spanish version developed by Bravo et al. [59]. The RTSQ is a 15-item self-report questionnaire rated on a seven-point Likert scale ranging from 1 (Not at all) to 7 (Very much). Higher scores indicate a greater tendency toward repetitive and recurrent ruminative thinking. The Spanish validation supported a four-factor structure comprising Problem-Focused Thoughts, Repetitive Thoughts, Anticipatory Thoughts, and Counterfactual Thinking, with good internal consistency and satisfactory evidence of construct validity [59]. The four RTSQ subscale scores were included in the statistical analyses.
6. Childhood Trauma Questionnaire—Short Form (CTQ-SF). Childhood maltreatment was assessed using the Childhood Trauma Questionnaire—Short Form (CTQ-SF) [60], employing the Spanish adaptation developed by Hernández et al. [61]. The CTQ-SF is a 28-item self-report questionnaire rated on a five-point Likert scale ranging from 1 (Never True) to 5 (Very Often True). It assesses five domains of childhood maltreatment: Emotional Abuse, Physical Abuse, Sexual Abuse, Physical Neglect, and Emotional Neglect. Higher total scores indicate greater exposure to childhood maltreatment. The Spanish version has demonstrated adequate internal consistency across its dimensions (Cronbach’s α = 0.66–0.94), and confirmatory factor analyses supported the original five-factor structure [61]. Only the CTQ-SF total score was included in the statistical analyses.
7. Symptom Checklist-90-Revised (SCL-90-R). General psychological symptomatology was assessed using the Symptom Checklist-90-Revised (SCL-90-R) [62]. The SCL-90-R is a 90-item self-report questionnaire rated on a five-point Likert scale ranging from 0 (Not at all) to 4 (Extremely), according to the degree of distress experienced during the previous week. The instrument assesses nine primary symptom dimensions, Somatization, Obsessive-Compulsive, Interpersonal Sensitivity, Depression, Anxiety, Hostility, Phobic Anxiety, Paranoid Ideation, and Psychoticism, and provides three global indices: the Global Severity Index (GSI), the Positive Symptom Total (PST), and the Positive Symptom Distress Index (PSDI) [62]. Only the GSI was included in the statistical analyses because the present study focused on overall psychological symptom severity rather than specific symptom domains. The GSI is calculated by summing the scores across the nine symptom dimensions and additional items and dividing this total by the number of completed items, with higher scores indicating greater overall psychological symptom severity [62].

The selection of total and subscale scores reflected the conceptual focus of the study. Dimensional scores were used for emotion-regulation and related emotional-cognitive constructs when the corresponding instruments assessed theoretically distinct processes relevant to trauma processing. The DERS total score was additionally included to contrast overall emotion-regulation difficulties with its specific dimensions. For childhood trauma and psychological symptomatology, global scores were used because these variables were conceptualized as overall trauma exposure and general symptom severity, respectively.

### 2.4. Data Analysis

Statistical analyses were conducted sequentially to examine the associations between emotional and cognitive variables, dimensions of the Processing Difficulties Scale (PDS), and general psychological symptom severity. First, descriptive statistics and zero-order Pearson correlations were calculated for the study variables. To account for multiple testing, the Benjamini–Hochberg false discovery rate (BH-FDR) procedure was applied globally to the 84 correlations between the 21 study variables and the four PDS dimensions, with the false discovery rate set at 5%. Associations with a BH-FDR-adjusted q < 0.05 were considered statistically significant.

Separate multivariable linear regression models were then estimated for the four PDS dimensions. Psychological predictors showing BH-FDR-adjusted associations with the corresponding PDS dimension were entered simultaneously (ENTER method), together with the number of EMDR processing sessions when its association with the corresponding PDS dimension survived BH-FDR correction. Although SCL-90-R GSI was included in the global BH-FDR correction, it was not entered as a predictor in these models because it was specified as the distal outcome in the subsequent indirect-effect analyses. This approach allowed the unique association of each predictor with the PDS dimension to be estimated while accounting for the other variables in the model. Regression diagnostics included variance inflation factors (VIFs) and tolerance values, assessment of residual normality, the Breusch–Pagan test for heteroscedasticity, Cook’s distance, leverage values, and studentized residuals. HC3 heteroscedasticity-consistent standard errors were also examined as a robustness check.

Potential dependence of observations within therapists was also examined. Intraclass correlation coefficients (ICCs) were estimated for the four PDS dimensions using random-intercept models. Given the limited number and unequal size of therapist clusters, the regression models were re-estimated as a sensitivity analysis using CR2 cluster-robust standard errors with Satterthwaite small-sample correction.

Because no formal a priori power analysis was conducted, a sensitivity analysis was performed to characterize the detectable effect size given the final analytical sample. For the most complex multivariable regression model (N = 116; eight predictors), the minimum detectable overall effect size was estimated at α = 0.05 and 80% statistical power.

Psychological predictors showing a statistically significant unique association (*p* < 0.05) in the regression models were subsequently examined in separate path models to assess potential indirect effects on general psychological symptom severity (SCL-90-R GSI) through the relevant PDS dimension. Direct paths from each predictor to GSI were retained. Given the cross-sectional design, these analyses were interpreted as indirect-effect models rather than as evidence of causal or temporal mediation.

Indirect effects were evaluated using 5000 nonparametric bootstrap resamples. Bias-corrected and accelerated 95% confidence intervals (BCa 95% CIs) were calculated, and an indirect effect was considered statistically significant when the confidence interval did not include zero. Analyses were conducted using R version 4.6.1 [63] and JASP version 0.98.0 [64].

**Figure 1:**
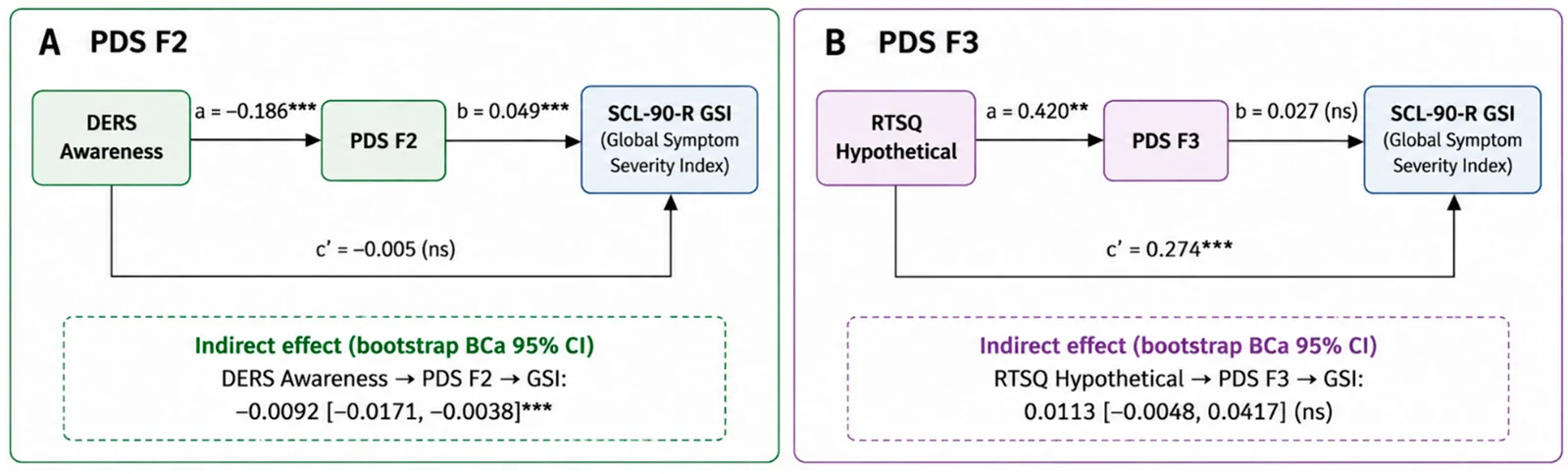
Focal indirect-effect models linking emotion regulation and repetitive thinking to general psychological symptom severity through PDS dimensions. Note. Values shown on paths are unstandardized coefficients (**B**). Indirect effects were estimated using 5000 nonparametric bootstrap resamples and are reported with bias-corrected and accelerated 95% confidence intervals (BCa 95% CIs). DERS = Difficulties in Emotion Regulation Scale; Awareness = lack of emotional awareness; RTSQ = Repetitive Thinking Styles Questionnaire; Hypothetical = hypothetical thoughts; PDS = Processing Difficulties Scale; F2 = Lack of Generalization and/or Absence of Changes; F3 = Poor Emotional Processing; SCL-90-R = Symptom Checklist-90-Revised; GSI = Global Severity Index. ** *p* < 0.01; *** *p* < 0.001; ns = not significant.

Figure 1 was prepared with the assistance of ChatGPT (OpenAI GPT-5.6 Sol) using the statistical results as input. The authors verified all paths, coefficients, significance levels, and confidence intervals against the original statistical output.

## 3. Results

The final analytical sample comprised 116 participants, including 93 women (80.2%) and 23 men (19.8%), with a mean age of 37.14 years (SD = 10.58). Regarding educational level, 5 participants (4.3%) had completed primary education, 23 (19.8%) secondary education, and 88 (75.9%) higher education. The most frequent diagnostic category was other diagnoses (31.9%), followed by anxiety disorders (28.4%), depressive disorders (23.3%), other trauma- and stressor-related disorders (9.5%), and post-traumatic disorders (6.9%).

Descriptive statistics and zero-order Pearson correlations between the study variables and the four PDS dimensions are presented in Table 1. Mean scores for the PDS dimensions were 11.21 (SD = 5.83) for PDS F1, 8.11 (SD = 5.20) for PDS F2, 5.17 (SD = 2.69) for PDS F3, and 3.37 (SD = 2.89) for PDS F4. The Benjamini–Hochberg false discovery rate (BH-FDR) correction was applied globally across the 84 correlations between the 21 study variables and the four PDS dimensions. Of the associations that were statistically significant at the unadjusted level, 21 remained significant after BH-FDR correction. The unadjusted *p* values and 95% confidence intervals corresponding to the Pearson correlations are reported below, while BH-FDR-adjusted significance is indicated in Table 1.

PDS F1 (Good Processing) was negatively associated with TAS-20 DIF, r(114) = −0.29, 95% CI [−0.45, −0.12], *p* = 0.002; TAS-20 DDF, r(114) = −0.30, 95% CI [−0.46, −0.12], *p* = 0.001; EPS-25 Difficulty, r(114) = −0.28, 95% CI [−0.44, −0.10], *p* = 0.002; RTSQ Problems, r(114) = −0.24, 95% CI [−0.40, −0.06], *p* = 0.010; and SCL-90-R GSI, r(114) = −0.32, 95% CI [−0.48, −0.15], *p* < 0.001.

PDS F2 (Lack of Generalization and/or Absence of Changes) was negatively associated with DERS Awareness, r(114) = −0.30, 95% CI [−0.46, −0.12], *p* = 0.001, and positively associated with DERS Nonacceptance, r(114) = 0.27, 95% CI [0.09, 0.43], *p* = 0.004; DERS Goals, r(114) = 0.28, 95% CI [0.11, 0.44], *p* = 0.002; DERS Strategies, r(114) = 0.30, 95% CI [0.13, 0.46], *p* < 0.001; TAS-20 DIF, r(114) = 0.28, 95% CI [0.10, 0.44], *p* = 0.003; EPS-25 Difficulty, r(114) = 0.24, 95% CI [0.06, 0.40], *p* = 0.010; EPS-25 Regulation, r(114) = 0.24, 95% CI [0.06, 0.40], *p* = 0.011; RTSQ Problems, r(114) = 0.32, 95% CI [0.15, 0.48], *p* < 0.001; and SCL-90-R GSI, r(114) = 0.35, 95% CI [0.18, 0.50], *p* < 0.001.

PDS F3 (Poor Emotional Processing) was positively associated with RTSQ Hypothetical, r(114) = 0.23, 95% CI [0.06, 0.40], *p* = 0.011.

PDS F4 (Loss of Dual Attention) was positively associated with EPS-25 Suppression, r(114) = 0.31, 95% CI [0.13, 0.47], *p* < 0.001; EPS-25 Difficulty, r(114) = 0.26, 95% CI [0.08, 0.42], *p* = 0.005; EPS-25 Regulation, r(114) = 0.26, 95% CI [0.08, 0.42], *p* = 0.004; EPS-25 Experience, r(114) = 0.27, 95% CI [0.10, 0.44], *p* = 0.003; SCL-90-R GSI, r(114) = 0.33, 95% CI [0.16, 0.48], *p* < 0.001; and EMDR Sessions, r(114) = 0.28, 95% CI [0.10, 0.44], *p* = 0.002. All remaining correlations are reported in Table 1.

Following the BH-FDR correction, separate multivariable linear regression models were estimated for each PDS dimension using the psychological predictors that remained significant in the corrected zero-order correlation analyses, together with EMDR processing sessions when its association with the corresponding PDS dimension survived BH-FDR correction. SCL-90-R GSI was not entered into these models because it was specified as the distal outcome in the subsequent indirect-effect analyses.

For PDS F1 (Good Processing), the model included TAS-20 DDF, TAS-20 DIF, EPS-25 Difficulty, and RTSQ Problems and was statistically significant, F(4, 111) = 4.65, *p* = 0.002, R^2^ = 0.144, adjusted R^2^ = 0.113. However, none of the individual predictor estimates provided clear evidence of a unique association with PDS F1 after accounting for the other predictors in the model, and all 95% confidence intervals included zero (Table 2).

For PDS F2 (Lack of Generalization and/or Absence of Changes), the overall model was statistically significant, F(8, 107) = 3.02, *p* = 0.004, R^2^ = 0.184, adjusted R^2^ = 0.123. DERS Awareness was the only predictor showing a statistically significant unique association with PDS F2, B = −0.135, SE = 0.060, β = −0.215, 95% CI [−0.255, −0.015], *p* = 0.028. The estimates for the remaining predictors were less precise and their 95% confidence intervals included zero (Table 2).

For PDS F3 (Poor Emotional Processing), RTSQ Hypothetical was the only predictor retained after BH-FDR correction and was positively associated with PDS F3, B = 0.420, SE = 0.163, β = 0.235, 95% CI [0.098, 0.742], *p* = 0.011. The overall model was statistically significant, F(1, 114) = 6.66, *p* = 0.011, R^2^ = 0.055, adjusted R^2^ = 0.047.

For PDS F4 (Loss of Dual Attention), the model included EPS-25 Suppression, EPS-25 Experience, EPS-25 Regulation, EPS-25 Difficulty, and EMDR processing sessions and was statistically significant, F(5, 110) = 4.75, *p* < 0.001, R^2^ = 0.177, adjusted R^2^ = 0.140. EMDR processing sessions was the only variable showing a statistically significant unique association with PDS F4, B = 0.043, SE = 0.015, β = 0.264, 95% CI [0.013, 0.072], *p* = 0.005. The estimates for the remaining predictors were less precise and their 95% confidence intervals included zero (Table 2).

Overall, the regression models explained 14.4% of the variance in PDS F1, 18.4% in PDS F2, 5.5% in PDS F3, and 17.7% in PDS F4. At the individual predictor level, the clearest unique associations were observed for DERS Awareness with PDS F2 (β = −0.215), RTSQ Hypothetical with PDS F3 (β = 0.235), and EMDR processing sessions with PDS F4 (β = 0.264). Full coefficient estimates and their 95% confidence intervals are reported in Table 2.

Regression diagnostics indicated no problematic multicollinearity across the models (maximum VIF = 3.21; minimum tolerance = 0.31). Shapiro–Wilk tests indicated departures from residual normality for PDS F1 (W = 0.977, *p* = 0.048) and PDS F4 (W = 0.934, *p* < 0.001), whereas no significant departures were observed for PDS F2 or F3. Breusch–Pagan tests provided no evidence of heteroscedasticity across the four models (all *p*s ≥ 0.124). No cases showed absolute studentized residuals greater than 3. Although Cook’s distance and leverage identified some potentially influential observations, HC3 heteroscedasticity-consistent standard errors yielded substantively unchanged conclusions.

The sensitivity analysis indicated that, with N = 116 and eight predictors, the minimum detectable overall effect size at α = 0.05 and 80% power was Cohen’s f^2^ = 0.14. As an additional robustness analysis, potential therapist-level clustering was examined. Therapist information was available for 115 participants nested within 13 therapists. The ICCs were 0.366 for PDS F1, 0.180 for PDS F2, 0.234 for PDS F3, and 0.109 for PDS F4, indicating varying degrees of therapist-level clustering across PDS dimensions. When the regression models were re-estimated using CR2 cluster-robust standard errors with Satterthwaite small-sample correction, coefficient estimates were essentially unchanged. The associations of RTSQ Hypothetical with PDS F3 (B = 0.420, SE = 0.123, *p* = 0.013) and EMDR processing sessions with PDS F4 (B = 0.043, SE = 0.013, *p* = 0.041) remained statistically significant. The estimate for DERS Awareness with PDS F2 was similar in magnitude to the primary analysis (B = −0.131, SE = 0.065), although less precise after accounting for therapist-level clustering (*p* = 0.122).

Two focal path models were estimated to examine potential indirect effects on general psychological symptom severity (SCL-90-R GSI) through the corresponding PDS dimensions. These models were restricted to psychological predictors that showed statistically significant unique associations with a PDS dimension in the preceding multivariable regression analyses (Table 3 and Figure 1).

For PDS F2 (Lack of Generalization and/or Absence of Changes), DERS Awareness was negatively associated with PDS F2 (B = −0.186, SE = 0.052, *p* < 0.001), and PDS F2 was positively associated with GSI after accounting for DERS Awareness (B = 0.049, SE = 0.013, *p* < 0.001). The indirect effect of DERS Awareness on GSI through PDS F2 was statistically significant (indirect effect = −0.0092, SE = 0.0033, 95% BCa bootstrap CI [−0.0171, −0.0038], *p* = 0.006). The direct association between DERS Awareness and GSI was not statistically significant (B = −0.005, *p* = 0.503).

For PDS F3 (Poor Emotional Processing), RTSQ Hypothetical was positively associated with PDS F3 (B = 0.420, SE = 0.161, *p* = 0.009), whereas PDS F3 was not significantly associated with GSI after accounting for RTSQ Hypothetical (B = 0.027, SE = 0.023, *p* = 0.246). Accordingly, the indirect effect of RTSQ Hypothetical on GSI through PDS F3 was not statistically significant (indirect effect = 0.0113, SE = 0.0110, 95% BCa bootstrap CI [−0.0048, 0.0417], *p* = 0.305). RTSQ Hypothetical retained a statistically significant direct association with GSI (B = 0.274, *p* < 0.001).

## 4. Discussion

The present study examined the associations between general difficulties in emotion regulation, specific regulatory and related emotional-cognitive processes, and patterns of trauma processing during EMDR therapy. Overall, the findings revealed differentiated patterns of association across the four PDS dimensions. Although several emotional and cognitive variables were associated with PDS dimensions at the bivariate level, these associations were substantially attenuated when predictors were considered simultaneously in the multivariable linear regression models. Significant independent associations were identified for Lack of Generalization and/or Absence of Changes (F2), Poor Emotional Processing (F3), and Loss of Dual Attention (F4), whereas no individual predictor remained independently associated with Good Processing (F1). Overall, these findings should be interpreted cautiously, as additional clinical and treatment-related factors are likely to contribute to individual differences in trauma processing.

A particularly relevant finding was that overall emotion regulation difficulties, as measured by the DERS total score, were not significantly associated with any of the four PDS dimensions. In contrast, several more specific regulatory and related emotional-cognitive processes, including emotional awareness, emotional processing difficulties, alexithymic traits, and repetitive negative thinking, showed differentiated bivariate associations with particular dimensions of trauma processing. This pattern suggests that examining specific regulatory and emotional-cognitive processes may provide more differentiated information about trauma processing than relying exclusively on a global index of emotion regulation difficulties. However, the attenuation of many of these associations in the multivariable analyses also indicates substantial overlap among these processes and cautions against interpreting their bivariate associations as independent contributions to trauma processing. This distinction is consistent with process-based approaches to psychopathology, which emphasize the examination of specific psychological processes beyond broad transdiagnostic constructs [14,19], and with previous evidence indicating that specific emotion regulation strategies and related processes may show differential associations with posttraumatic symptoms and trauma adaptation [65,66,67].

The absence of significant associations between the EPS-25 Avoidance subscale and any of the PDS dimensions after correction for multivariable comparisons is also noteworthy. One possible explanation may relate to how avoidance was operationalized in the present study. The EPS-25 Avoidance subscale primarily assesses behavioral avoidance in the context of emotional processing [55] and should not necessarily be considered equivalent to the broader construct of experiential avoidance, which refers to attempts to avoid or alter unwanted internal experiences [15,37]. Accordingly, the present findings should not be interpreted as indicating that avoidance is unrelated to trauma-related psychological functioning, but rather that the specific behavioral avoidance tendencies assessed by the EPS-25 were not significantly associated with patterns of trauma processing during EMDR in this diagnostically heterogeneous clinical sample.

For Good Processing (F1), none of the variables included in the model showed a significant independent association, despite the bivariate associations observed with difficulties describing and identifying emotions, emotional processing difficulties, problem-focused thinking, and overall symptom severity. This pattern suggests that variability in Good Processing may reflect the combined contribution of multiple overlapping processes or other factors not examined here. In contrast, lower emotional awareness remained independently associated with greater Lack of Generalization and/or Absence of Changes (F2). Difficulties attending to and recognizing emotional experience may be particularly relevant to the integration and updating of emotional information during trauma processing, although the cross-sectional nature of the present findings does not allow the direction of this association to be established [22,23,24].

Poor Emotional Processing (F3) showed a more specific pattern, with hypothetical thinking emerging as its only significant independent correlate. A greater tendency to engage in hypothetical repetitive thinking was associated with greater difficulties in emotional processing during trauma reprocessing. This finding may be consistent with evidence suggesting that repetitive negative thinking can maintain attention on internally generated cognitive content and interfere with flexible emotional processing [33,34]. However, the model accounted for only 5.5% of the variance in F3, indicating that hypothetical thinking represents, at most, a limited correlate of this processing pattern and that other unmeasured factors are likely to be involved. For Loss of Dual Attention (F4), emotional suppression and several other dimensions of emotional processing were associated with greater processing difficulties at the bivariate level. However, these associations were attenuated in the multivariable model, in which the number of EMDR processing sessions was the only variable that remained independently associated with F4. This association should be interpreted cautiously. Rather than indicating that a greater number of sessions contributes to loss of dual attention, patients with greater clinical complexity or more persistent difficulties during trauma processing may require a larger number of processing sessions. Because the cross-sectional design does not establish temporal ordering, the direction of this association cannot be determined, and prospective studies are needed to clarify the relationship between treatment duration and difficulties maintaining dual attention during EMDR processing.

The cross-sectional indirect-effect analyses provided further evidence of differentiated associations across PDS dimensions. A significant indirect association was observed between lower emotional awareness and greater overall psychological symptom severity through Lack of Generalization and/or Absence of Changes (F2), whereas the indirect effect of hypothetical thinking on symptom severity through Poor Emotional Processing (F3) was not significant. The significant F2 indirect effect is compatible with the possibility that difficulties in attending to emotional experience and difficulties in integrating or generalizing information during trauma processing are jointly related to broader psychological distress. This interpretation is broadly consistent with theoretical accounts emphasizing emotional processing and the updating and integration of emotional information during trauma-focused treatment [23,24,28]. However, because all variables were assessed within a cross-sectional design, the indirect effect represents a statistical decomposition of associations and does not establish temporal ordering or a mediating mechanism. Longitudinal studies are therefore required to determine whether emotional awareness prospectively predicts subsequent difficulties in trauma processing and changes in psychological symptom severity.

The present findings may also be considered in the context of the ongoing debate regarding the extent of preparation or stabilization needed before trauma-focused processing in individuals with complex clinical presentations. Whereas phase-based approaches emphasize the potential value of addressing emotion regulation difficulties before initiating trauma processing [43], other evidence suggests that trauma-focused interventions can be implemented without extensive prior stabilization [44,45]. The present cross-sectional findings do not support either position or establish that the emotional and cognitive processes identified here should be targeted before memory reprocessing. Rather, they generate hypotheses about whether specific processes, such as emotional awareness or repetitive thinking, may be relevant to individual differences in trauma processing and warrant prospective investigation as potential targets for individualized preparation or support during EMDR therapy.

Finally, these findings should be interpreted in light of several limitations. The cross-sectional design precludes causal and temporal inferences, particularly regarding the indirect-effect analyses. No formal a priori power analysis was conducted, and the available sample size may have limited the ability to detect smaller effects and the precision of some estimates. Accordingly, effect sizes and their confidence intervals should be considered alongside statistical significance when interpreting the findings. Moreover, the modest variance explained by the multivariable models indicates that other patient-, therapist-, and treatment-related factors are likely to contribute to trauma processing. The diagnostically heterogeneous sample limits conclusions regarding specific diagnostic populations. In addition, the nature and type of traumatic memories being reprocessed were not systematically recorded, limiting the characterization of trauma exposure beyond the available childhood trauma measure. The retrospective assessment of the PDS by treating therapists may be susceptible to recall and observer biases, and independent ratings of the same patients were not available, precluding the assessment of interrater reliability. Some therapist-level clustering was also observed in PDS scores. Although sensitivity analyses accounting for therapist clustering yielded largely similar coefficient estimates, the small number and unequal size of therapist clusters reduced the precision of some cluster-adjusted estimates. Although all participating therapists were EMDR Clinicians using the standard EMDR protocol, formal treatment-fidelity ratings were not collected, limiting the ability to assess variability in protocol implementation across therapists. Finally, the absence of longitudinal assessments prevents determining whether the observed processing patterns predict subsequent treatment response. Future prospective studies with larger samples and repeated assessments are needed to replicate these findings, establish their temporal relationships with treatment outcomes, and examine the predictive and clinical utility of the PDS across diagnostic groups and treatment settings.

## 5. Conclusions

The present study provides preliminary evidence that patterns of trauma processing during EMDR therapy are differentially associated with specific regulatory and emotional-cognitive processes. Whereas overall emotion regulation difficulties were not significantly associated with the PDS dimensions, several specific processes showed differentiated bivariate associations, including an association between emotional suppression and Loss of Dual Attention, although many of these associations were attenuated after simultaneous adjustment. In the multivariable analyses, lower emotional awareness was independently associated with greater Lack of Generalization and/or Absence of Changes, hypothetical thinking with greater Poor Emotional Processing, and the number of EMDR processing sessions with greater Loss of Dual Attention.

The cross-sectional indirect-effect analyses further identified a significant statistical indirect association between lower emotional awareness and greater psychological symptom severity through Lack of Generalization and/or Absence of Changes, whereas no corresponding indirect association was observed for hypothetical thinking through Poor Emotional Processing. These findings highlight the potential value of examining differentiated patterns of trauma processing and specific emotional-cognitive processes, while the cross-sectional design and modest explanatory capacity of the models preclude conclusions regarding temporal or causal mechanisms.

Future prospective studies with repeated assessments are needed to determine whether these processes and processing patterns predict subsequent treatment response and to clarify their temporal relationships throughout EMDR therapy. Such research may contribute to a more precise understanding of individual differences in trauma processing and establish whether the PDS can provide clinically useful information about processing patterns and treatment outcomes.

## Figures and Tables

**Table 1:** Zero-order correlations between study variables and PDS dimensions.

Variable	M	SD	PDS F1	PDS F2	PDS F3	PDS F4
CTQ Total	51.07	14.42	−0.07	−0.02	0.22	0.12
DERS Awareness	15.16	8.29	0.02	−0.30 *	0.13	−0.15
DERS Clarity	10.40	3.47	−0.18	−0.02	0.17	0.01
DERS Nonacceptance	17.22	7.61	−0.05	0.27 *	−0.05	0.08
DERS Goals	11.85	4.50	−0.19	0.28 *	0.05	0.12
DERS Strategies	19.22	6.98	−0.21	0.30 *	0.09	0.12
DERS Total	59.09	16.92	−0.11	−0.04	0.17	−0.02
TAS-20 DIF	20.61	9.71	−0.29 *	0.28 *	0.17	0.15
TAS-20 DDF	15.11	6.04	−0.30 *	0.20	0.06	0.07
TAS-20 EOT	17.55	6.11	−0.08	0.21	0.02	0.04
EPS-25 Suppression	4.20	2.15	−0.22	0.19	0.22	0.31 **
EPS-25 Difficulty	4.55	2.09	−0.28 *	0.24 *	0.17	0.26 *
EPS-25 Regulation	2.98	2.13	−0.13	0.24 *	0.13	0.26 *
EPS-25 Avoidance	4.34	2.19	−0.11	0.17	0.06	0.19
EPS-25 Experience	2.52	1.94	−0.15	0.15	0.18	0.27 *
RTSQ Repetitive	4.95	1.52	−0.20	0.22	0.13	0.07
RTSQ Hypothetical	4.22	1.51	−0.19	0.22	0.23 *	0.14
RTSQ Problems	4.06	1.73	−0.24 *	0.32 **	0.09	0.16
RTSQ Anticipatory	4.50	1.50	−0.07	0.07	0.10	0.21
SCL-90-R GSI	1.41	0.76	−0.32 **	0.35 **	0.22	0.33 **
EMDR Sessions	15.88	17.87	−0.17	0.20	−0.07	0.28 *

Note. M = mean; SD = standard deviation. Values are Pearson’s r. The Benjamini–Hochberg false discovery rate (BH-FDR) correction was applied globally across the 84 correlations between the 21 study variables and the four PDS dimensions. * q < 0.05; ** q < 0.01. PDS = Processing Difficulties Scale; F1 = Good Processing; F2 = Lack of Generalization and/or Absence of Changes; F3 = Poor Emotional Processing; F4 = Loss of Dual Attention. CTQ = Childhood Trauma Questionnaire; Total = total childhood trauma score. DERS = Difficulties in Emotion Regulation Scale; Awareness = lack of emotional awareness; Clarity = lack of emotional clarity; Nonacceptance = nonacceptance of emotional responses; Goals = difficulties engaging in goal-directed behavior; Strategies = difficulties in emotion regulation; Total = total difficulties in emotion regulation score. TAS-20 = 20-item Toronto Alexithymia Scale; DIF = Difficulty Identifying Feelings; DDF = Difficulty Describing Feelings; EOT = Externally Oriented Thinking. EPS-25 = Emotional Processing Scale; Suppression = emotional suppression; Difficulty = signs of unprocessed emotion; Regulation = emotional control; Avoidance = emotional avoidance; Experience = emotional experience. RTSQ = Repetitive Thinking Styles Questionnaire; Repetitive = repetitive thoughts; Hypothetical = hypothetical thoughts; Problems = problem-focused thoughts; Anticipatory = anticipatory thoughts. SCL-90-R = Symptom Checklist-90-Revised; GSI = Global Severity Index. EMDR = Eye Movement Desensitization and Reprocessing; EMDR sessions = number of EMDR sessions involving trauma memory reprocessing.

**Table 2:** Multivariable linear regression models predicting PDS dimensions.

Outcome/Model	Predictor	B	SE	β	95% CI for B	*p*	R^2^	Adjusted R^2^
PDS F1	TAS-20 DDF	−0.193	0.098	−0.200	[−0.388, 0.002]	0.052	0.144	0.113
	TAS-20 DIF	−0.076	0.066	−0.127	[−0.207, 0.055]	0.253		
	EPS-25 Difficulty	−0.508	0.311	−0.182	[−1.124, 0.108]	0.105		
	RTSQ Problems	0.058	0.401	0.017	[−0.737, 0.853]	0.886		
PDS F2	RTSQ Problems	0.367	0.390	0.122	[−0.406, 1.139]	0.349	0.184	0.123
	DERS Strategies	0.003	0.100	0.004	[−0.196, 0.201]	0.978		
	DERS Awareness	−0.135	0.060	−0.215	[−0.255, −0.015]	0.028		
	DERS Goals	0.186	0.134	0.161	[−0.079, 0.451]	0.168		
	TAS-20 DIF	0.065	0.058	0.121	[−0.049, 0.179]	0.263		
	DERS Nonacceptance	−0.031	0.088	−0.046	[−0.207, 0.144]	0.725		
	EPS-25 Difficulty	−0.061	0.322	−0.024	[−0.698, 0.577]	0.850		
	EPS-25 Regulation	0.188	0.287	0.077	[−0.381, 0.757]	0.513		
PDS F3	RTSQ Hypothetical	0.420	0.163	0.235	[0.098, 0.742]	0.011	0.055	0.047
PDS F4	EPS-25 Suppression	0.159	0.134	0.119	[−0.106, 0.424]	0.239	0.177	0.140
	EPS-25 Experience	0.225	0.210	0.151	[−0.192, 0.642]	0.287		
	EMDR Sessions	0.043	0.015	0.264	[0.013, 0.072]	0.005		
	EPS-25 Regulation	0.052	0.183	0.038	[−0.310, 0.414]	0.777		
	EPS-25 Difficulty	0.083	0.175	0.060	[−0.264, 0.429]	0.638		

Note. B = unstandardized regression coefficient; SE = standard error; β = standardized regression coefficient; CI = confidence interval; R^2^ = coefficient of determination. Predictors retained after the global BH-FDR correction were entered simultaneously into each model. PDS = Processing Difficulties Scale; F1 = Good Processing; F2 = Lack of Generalization and/or Absence of Changes; F3 = Poor Emotional Processing; F4 = Loss of Dual Attention. TAS-20 = 20-item Toronto Alexithymia Scale; DDF = Difficulty Describing Feelings; DIF = Difficulty Identifying Feelings. EPS-25 = Emotional Processing Scale; Difficulty = signs of unprocessed emotion; Regulation = emotional control; Suppression = emotional suppression; Experience = emotional experience. RTSQ = Repetitive Thinking Styles Questionnaire; Problems = problem-focused thoughts; Hypothetical = hypothetical thoughts. DERS = Difficulties in Emotion Regulation Scale; Strategies = difficulties in emotion regulation; Awareness = lack of emotional awareness; Goals = difficulties engaging in goal-directed behavior; Nonacceptance = nonacceptance of emotional responses. EMDR sessions = number of EMDR sessions involving trauma memory reprocessing.

**Table 3:** Focal indirect-effect models predicting general psychological symptom severity.

Model	Predictor (X)	Mediator (M)	a: X → M B (SE), *p*	b: M → GSI B (SE), *p*	c′: X → GSI B, *p*	Indirect Effect ab (SE), *p*	95% BCa Bootstrap CI
F2	DERS Awareness	PDS F2	−0.186 (0.052), <0.001	0.049 (0.013), <0.001	−0.005, 0.503	−0.0092 (0.0033), 0.006	[−0.0171, −0.0038]
F3	RTSQ Hypothetical	PDS F3	0.420 (0.161), 0.009	0.027 (0.023), 0.246	0.274, <0.001	0.0113 (0.0110), 0.305	[−0.0048, 0.0417]

Note. B = unstandardized coefficient; SE = standard error; a = path from predictor to mediator; b = path from mediator to SCL-90-R GSI controlling for the predictor; c′ = direct effect of the predictor on SCL-90-R GSI controlling for the mediator; BCa = bias-corrected and accelerated. Indirect effects were estimated using 5000 nonparametric bootstrap resamples and were considered statistically significant when the 95% BCa bootstrap confidence interval did not include zero. DERS = Difficulties in Emotion Regulation Scale; Awareness = lack of emotional awareness; RTSQ = Repetitive Thinking Styles Questionnaire; Hypothetical = hypothetical thoughts; PDS = Processing Difficulties Scale; F2 = Lack of Generalization and/or Absence of Changes; F3 = Poor Emotional Processing; SCL-90-R = Symptom Checklist-90-Revised; GSI = Global Severity Index.

## Data Availability

The data used in this study are available upon request from the corresponding author. However, data sharing is subject to Research Ethics Committee approval and applicable data protection regulations.
